# Lifestyle factors are significantly associated with the locomotive syndrome: a cross-sectional study

**DOI:** 10.1186/s12877-017-0630-1

**Published:** 2017-10-18

**Authors:** Manabu Akahane, Shingo Yoshihara, Akie Maeyashiki, Yasuhito Tanaka, Tomoaki Imamura

**Affiliations:** Department of Public Health, Health Management and Policy, Nara Medical University Faculty of Medicine, Shijo 840, Kashihara, Nara Japan

**Keywords:** Lifestyle, Musculoskeletal disease, Locomotive, Smoking, Drinking, Periodontal disease, Tooth, Risk reduction behavior

## Abstract

**Background:**

The Japanese Orthopedic Association first proposed the concept of “locomotive syndrome” in 2007. It refers to circumstances in which elderly people need nursing care services or are at high risk of requiring such services within a short time. Recently, the public health burden of providing nursing care for elderly individuals has increased. Therefore, locomotive syndrome, and the means of preventing it, are a major public health focus in Japan. The purpose of this study was to investigate the relationships of lifestyle factors, such as smoking, alcohol consumption, sleep duration, and dental health, with locomotive syndrome.

**Methods:**

We conducted a cross-sectional study using an internet panel survey. The participants comprised 747 individuals aged 30–90 years. Factors related to demographics (age, sex), general health (number of teeth, presence of periodontal disease), and lifestyle (smoking, alcohol consumption, sleep duration) were assessed. We also used the 25-question Geriatric Locomotive Function Scale to determine whether each participant had locomotive syndrome. Multivariate analysis was conducted using logistic regression to investigate the independent relationships between locomotive syndrome and lifestyle factors after adjusting for sex and age.

**Results:**

A greater proportion of women (17.7%) than men (11.2%) had locomotive syndrome (*p* < 0.05). Participants aged ≥65 years showed significantly higher percentages (men: 21.4%, women: 75.7%) of locomotive syndrome compared with those aged <65 years (p < 0.05). Logistic regression analysis revealed that older age (≥ 65 years), sex, current smoking status, number of existing teeth, and presence of periodontal disease were associated with locomotive syndrome, whereas sleep duration was not. The frequency of alcohol consumption, except for daily drinking, was also associated with locomotive syndrome.

**Conclusion:**

Our study indicates that lifestyle factors, such as smoking and number of existing teeth, may partly affect the prevalence of locomotive syndrome. Hence, lifestyle modifications, such as improving oral hygiene and promoting cessation of smoking, are important means to reduce the risk of locomotive syndrome and should be promoted by public health staff.

## Background

The Japanese Orthopedic Association first proposed the concept of “locomotive syndrome” in 2007. It refers to circumstances in which elderly individuals need nursing care services or are at high risk of requiring such services within a short period of time [1]. To recognize whether an individual is at risk of locomotive syndrome, one can use a self-administered checklist called the “loco-check,” which consists of seven items regarding daily activities and is an acceptable measure for detecting early-stage locomotive syndrome [1]. It is simple and easy to understand even for elderly individuals, since the seven daily activities represent gait and balance abilities. Recently, the 25-question Geriatric Locomotive Function Scale (GLFS-25) was developed as a screening measure for detecting locomotive syndrome [2]. This scale comprises 25 items covering daily activities and symptoms involving locomotive organs, such as pains of the knees, hips, and spine. The cutoff score for identifying locomotive syndrome is 16/100; an individual who scores ≥16 is considered as high-risk and may soon require care services if no medical intervention is not provided.

Researchers have reported that physical factors, such as knee and spinal factors—including back muscle strength and low back pain—are related to locomotive syndrome [3–5]. Decreasing back muscle strength and aging are reported to be the most important risk factors for locomotive syndrome [4]. Others have reported that central obesity is associated with the risk of locomotive syndrome and that waist circumference could be a useful parameter to assess the risk of locomotive syndrome in elderly women [6]. Locomotive syndrome strongly impacts the individual’s quality of life and appears to be a useful concept for screening individuals with low quality of life due to musculoskeletal diseases, such as lumbar disease and knee osteoarthritis (OA) [7]. However, few reports have assessed the relationship between locomotive syndrome and lifestyle factors, such as smoking and alcohol consumption. Accordingly, it is still unclear whether lifestyle factors affect the development of locomotive syndrome.

Various lifestyle factors, including lack of exercise, smoking, alcohol consumption, obesity, and mental stress have been demonstrated to be related to increasing risks of diseases such as heart disease, stroke, and type II diabetes. Although these lifestyle factors are important individual risk factors for the progression of various diseases, combinations of these factors have also been shown to have a significant impact on several diseases. Because the public health burden of providing nursing care for elderly people is rising, studies on locomotive syndrome and preventive care are a major public health focus in Japan. The aim of the present study was to investigate the relationships of lifestyle factors, such as smoking, alcohol consumption, sleep duration, and dental health (number of teeth and periodontal disease) with locomotive syndrome.

## Methods

### Study design

The general health of the participants, including lifestyle factors and GLFS-25 data, was investigated in April 2011. We conducted a cross-sectional study using an internet panel survey. All participants were recruited by a panel survey company. The participants of the present study included registered panel members aged between 30 and 90 years. First, to recruit the participants for the present study, the survey company created a list by random sampling from all registers. Next, an email asking whether they were interested in participating in our survey was sent to all individuals on this list. The registration was closed when the number of participants in each group reached the target sample size. The participants completed and transmitted their responses via mail. After the completion of the survey, the participants received a small cash reward. After data cleaning, the included participants (*n* = 747) were stratified into three age groups: 30–49 years (*n* = 248), 50–64 years (*n* = 202), and 65–90 years (*n* = 297). There were 374 and 373 male and female participants, respectively.

### Survey of lifestyle and health condition

The age, sex, weight, height, and residential area were already recorded during the registration of the members to the survey panel. Next, the survey enquired about lifestyle factors and administered the GLFS-25 questionnaire, as detailed below.

We asked about lifestyle factors, including smoking, alcohol consumption, and sleep duration. The smoking status was classified as non-smokers, past smokers, and current smokers. Alcohol consumption was categorized according to the frequency of drinking using a multiple-choice item with four response options: none, a few times/month, a few times/week, and (almost) daily. The sleep duration per 24-h period was also a multiple-choice item, with three response options (≤ 6 h, 7 h, or ≥8 h). Further, we asked about the participants’ dental health in terms of the number of teeth present, including artificial teeth (dental implants), and the presence of periodontal disease. The numbers of existing teeth were categorized as ≤9, 10–19, 20–27, and ≥28.

### Self-assessment using the GLFS-25 questionnaire

The GLFS-25 questionnaire was developed as a screening tool to detect individuals at high risk of conditions that may soon require care services [2]. This is a self-administered questionnaire consisting of 25 items: 4 items for pain, 16 for activities of daily living, 3 for social functions, and 2 for the mental health status during the last month [2]. Each item is scored from 0 (no impairment) to 4 (severe impairment) points. The total score ranges from 0 to 100 points. Seichi et al. proposed that an individual whose GLFS-25 score is ≥16 points can be diagnosed as having locomotive syndrome [2] [8]. Thus, in the present study, locomotive syndrome was considered to be present in cases of GLFS-25 ≥ 16 points.

### Statistical analysis

Differences in age, number of teeth present, the presence of periodontal disease, and smoking were compared using the Chi-square test or *t*-test, as appropriate. A cross table was created with items such as the age category, number of teeth, the presence of periodontal disease, smoking, alcohol consumption, and sleep duration to show the distributions of participant characteristics according to sex and the presence of locomotive syndrome.

Logistic regression analysis was conducted with the presence or absence of locomotive syndrome as the dependent variable. The independent variables included sex, age category, smoking, frequency of alcohol consumption, sleep duration, number of existing teeth, and the presence of periodontal disease. All statistical analyses were conducted using SPSS version 21.0 (IBM, Chicago, IL, USA). The level of significance was set at *p* < 0.05.

## Results

### Baseline characteristics

The mean (± standard deviation) ages were 58.7 ± 16.6 and 58.8 ± 16.9 years for male and female participants, respectively. The mean numbers of teeth present were 23.1 ± 7.9 and 23.2 ± 8.0, respectively, in male and female participants (*p* > 0.05), and the prevalence rates of periodontal disease were 19.3% and 18.4%, respectively (*p* > 0.05). A significantly greater proportion of male than female participants smoked (20.6% vs. 6.7%, *p* < 0.01).

### Proportions of participants with locomotive syndrome

Locomotive syndrome was identified in 11.2% and 17.7% of male and female participants, respectively (*p* < 0.05). A significantly greater proportion of individuals aged ≥65 years had locomotive syndrome compared with those aged <65 years (*p* < 0.05). Among the group aged ≥65 years, there was a significant difference in the prevalence of locomotive syndrome between male and female participants (21.4% vs. 75.7%, *p* < 0.05).

### Distributions of participant characteristics according to sex and the presence of locomotive syndrome

The distributions of the participant characteristics according to sex and the presence or absence of locomotive syndrome are shown in Table 1. Participants aged ≥65 years tended to have locomotive syndrome more frequently, especially among female participants. Participants with locomotive syndrome tended to have fewer teeth and to more frequently have periodontal disease.

**Table 1:** Distribution of the participant characteristics according to sex and presence of locomotive syndrome

	Males				Females				Total			
	Locomotive syndrome				Locomotive syndrome				Locomotive syndrome			
	No		Yes		No		Yes		No		Yes	
	n	%	n	%	n	%	n	%	n	%	n	%
*Age category (years)*												
30–64	211	63.6	9	21.4	215	70.0	15	22.7	426	66.7	24	22.2
≥ 65	121	36.4	33	78.6	92	30.0	51	77.3	213	33.3	84	77.8
*Number of teeth*												
≤ 9	28	8.5	8	19.5	14	4.6	22	34.9	42	6.6	30	28.8
10–19	37	11.2	9	22.0	25	8.2	12	19.0	62	9.8	21	20.2
20–27	115	35.0	17	41.5	113	37.0	17	27.0	228	36.0	34	32.7
≥ 28	149	45.3	7	17.1	153	50.2	12	19.0	302	47.6	19	18.3
*Periodontal disease*												
Absent	252	82.1	28	70.0	235	85.1	36	64.3	487	83.5	64	66.7
Present	55	17.9	12	30.0	41	14.9	20	35.7	96	16.5	32	33.3
*Smoking*												
None	139	41.9	16	38.1	259	84.4	51	77.3	398	62.3	67	62.0
Past smoker	124	37.3	18	42.9	31	10.1	7	10.6	155	24.3	25	23.1
Current smoker	69	20.8	8	19.0	17	5.5	8	12.1	86	13.5	16	14.8
*Alcohol consumption*												
None	64	19.3	19	45.2	120	39.1	39	59.1	184	28.8	58	53.7
A few times/month	63	19.0	2	4.8	80	26.1	10	15.2	143	22.4	12	11.1
A few times/week	105	31.6	7	16.7	72	23.5	12	18.2	177	27.7	19	17.6
Daily	100	30.1	14	33.3	35	11.4	5	7.6	135	21.1	19	17.6
*Sleep duration* (per 24 h)												
≤ 6 h	160	48.2	17	41.5	152	49.5	25	37.9	312	48.8	42	39.3
7 h	99	29.8	9	22.0	99	32.2	15	22.7	198	31.0	24	22.4
≥ 8 h	73	22.0	15	36.6	56	18.2	26	39.4	129	20.2	41	38.3

### Relationship between locomotive syndrome and lifestyle factors

The results of the age- and lifestyle-adjusted logistic regression analysis are shown in Table 2. A particularly high odds ratio was found for age ≥ 65 years. Sex, current smoking, number of existing teeth, and presence of periodontal disease were associated with locomotive syndrome, whereas sleep duration was not. The frequency of alcohol consumption, except for daily drinking, was also associated with locomotive syndrome.

**Table 2:** Logistic regression analysis of the associations between lifestyle factors and locomotive syndrome

	OR	95% CI		*p* value
		Lower	Upper	
*Sex*				
Male	ref			
Female	2.28	1.26	4.12	0.01
*Age category (years)*				
30–64	ref			
≥ 65	5.12	2.83	9.29	<0.001
*Number of teeth category*				
≤ 9	ref			
10–19	0.57	0.25	1.27	0.17
20–27	0.43	0.21	0.89	0.02
≥ 28	0.28	0.13	0.63	<0.01
*Periodontal disease*				
Absent	ref			
Present	1.96	1.13	3.41	0.02
*Smoking*				
None	ref			
Past smoker	1.46	0.74	2.89	0.27
Current smoker	2.45	1.10	5.42	0.03
*Alcohol consumption*				
None	ref			
A few times/month	0.48	0.22	1.05	0.07
A few times/week	0.48	0.24	0.96	0.04
Daily	0.61	0.30	1.22	0.16
*Sleep duration* (per 24 h)				
≤ 6 h	ref			
7 h	0.84	0.46	1.57	0.59
≥ 8 h	1.41	0.78	2.53	0.26

*OR* odds ratio, *CI* confidence interval

## Discussion

Our study showed that older age (≥ 65 years), sex, current smoking status, number of existing teeth, presence of periodontal disease, and the frequency of alcohol consumption were associated with locomotive syndrome, whereas sleep duration was not. Of these factors, the frequency of alcohol consumption, except for daily drinking, and number of existing teeth showed inverse relationships with locomotive syndrome. To our knowledge, this is the first study to show an association between locomotive syndrome and lifestyle factors such as smoking and number of existing teeth. Lifestyle modifications, such as improving oral hygiene and promoting the cessation of smoking, may represent important means to reduce the risk of locomotive syndrome.

It is well known that various lifestyle factors, including smoking, alcohol consumption, obesity, lack of physical exercise, and stress, are associated with increasing risks of certain diseases, designated as lifestyle-related diseases. In particular, smoking is a widespread public health problem; it has been shown to be associated with not only cancer, diabetes, and cardiovascular diseases [5], but also with chronic musculoskeletal disorders such as OA, back pain, sciatic pain, and intervertebral disc disorders [9–11]. OA in the knee joint and spinal disorders are important disorders related to locomotive syndrome [12, 13]. It has been reported that men with knee OA who smoke sustain greater cartilage loss and have more severe knee pain than men who do not smoke [10]. OA, which carries a large community health burden, is a common, complex disorder with various risk factors. The symptoms of OA are related to cartilage degradation, which involves synovial inflammation, subchondral bone remodeling, and osteophyte formation. Therefore, current smoking may be a crucial and modifiable determinant of locomotive syndrome and lifestyle-related diseases. Hence, promoting cessation of smoking may decrease the proportion of the population who require nursing care services.

The dental health status in older adults has been associated with physical fitness [14–16]. Deterioration of dental health is not only related to oral function but also to general health among elderly individuals. Several reports have described the influence of oral health on motor performance and muscle strength of the extremities [17–20] and on mortality [21]. Moriya et al. [20] reported that the self-assessed masticatory ability may be significantly related to muscle strength and static balance function. Gaszynska et al. [19] reported that there is a relationship between chewing ability and physical fitness in elderly individuals. Taken together, these studies suggest that physical performance is significantly associated with dental status and periodontal status, as well as chewing ability, among the elderly. Others have reported that nutritional status is associated with dentition status [22, 23]. Sheiham et al. [22] conducted a cross-sectional survey using part of the nationwide British National Diet and Nutrition Survey, which showed that the dental status of older people associated with their ability to eat, affecting the food choice and preparation, and ultimately the intake and blood levels of some key nutrients. Our study observed that the number of existing teeth and the presence of periodontal disease related to the presence of locomotive syndrome. Interestingly, the number of existing teeth showed an inverse relationship with the presence of locomotive syndrome, indicating that dental health workers could contribute to strategies for preventing locomotive syndrome. Proper and continuous health education from an early age may be one important strategy to maintain one’s dental health and ensure good nutritional status.

Several reports have described the influence of alcohol intake on bone strength [24–26] and bone remodeling [27, 28]. Gino et al. [24] reported that light to moderate alcohol consumption was beneficial, resulting in higher bone mineral density and reduced age-related bone loss. However, heavy alcohol consumption was found to be associated with decreased bone mineral density, impaired bone quality, and increased fracture risk. Osteoporosis, which is one potential cause of locomotive syndrome, is associated with a number of lifestyle factors, including nutritional status and alcohol consumption [25]. Moderate alcohol consumption has been shown to have a protective effect against osteoporosis; however, heavy alcohol intake conversely associates with an increased risk of osteoporosis [25]. Maurel et al. [29] reported that the decrease in bone mass and strength following alcohol consumption is mainly due to a bone remodeling imbalance, with a predominant decrease in bone formation. They also concluded that the effects of alcohol consumption on bone were linked to the dose ingested and the duration of consumption. Consumption of one drink per day for women and two for men was not deleterious for bone tissue, whereas higher consumption such as 2–4 drinks per day was found to relate to damage of the bone tissue. Our study observed that alcohol consumption a few times/month or a few times/week inversely related to the presence of locomotive syndrome. This may indicate that light to moderate alcohol consumption is beneficial in relation to the risk of locomotive syndrome, although the dose ingested and the duration of alcohol consumption were not assessed in the present study.

Insomnia and disturbed sleep are increasingly common among older people [30]. Although sleep disturbance causes slowed responses and subsequently results in a greater risk of accidents, which may lead to locomotive syndrome, we did not find an association between sleep duration and locomotive syndrome in this study. Our participants were generally sleeping more than 6 h per night, indicating that our sample generally slept well.

Finally, our study indicated that older age (≥ 65 years) significantly associated with the risk of locomotive syndrome, as compared to younger age (< 65 years), consistent with previous reports [2, 7, 31, 32]. Nevertheless, although the prevalence of locomotive syndrome was low, some participants aged <65 years also had locomotive syndrome in our study. Seichi et al. [31] reported that the prevalence rates of locomotive syndrome in Japan were 4.6%, 7.8%, 12.0%, and 24.5% for individuals in their 40s, 50s, 60s, and 70s, respectively, indicating a gradual increase with advancing age. They also estimated that the total number of individuals with locomotive syndrome aged between 40 and 79 years was approximately 7.5 million. Taken together, these data, as well as the results from the present study, indicate that public health staff need to take measures to prevent locomotive syndrome in high-risk individuals, even when relatively young. In Japan, individuals aged ≥65 years accounted for 26.0% of the population in 2014; this characterizes the country as a “super aging society” [33]. The percentage of individuals aged ≥65 years is estimated to reach approximately 30% and 40% in 2025 and 2060, respectively [33]. Similarly, the percentages of individuals aged ≥65 years in developed countries other than Japan are also increasing. Accordingly, the prevalence of locomotive syndrome will likely increase in the near future, since aging is related to increased risks of musculoskeletal problems such as spinal disorders and OA in the knee and hip joints [34, 35], thereby increasing the need for nursing care services. Our study indicated that the risk of locomotive syndrome could potentially be decreased by instituting lifestyle modifications such as changing diet, increasing exercise, improving oral hygiene, and promoting the cessation of smoking. Public health staff can, and should, help promote such interventions.

This study has some limitations that must be acknowledged. First, we used an internet panel survey to collect the data. Thus, all measures were based on self-report, and this may result in some misclassification. Second, we collected data from respondents aged between 30 and 90 years who were registered with an internet panel company. Elderly individuals who use computers and the internet may be more active and healthier than those who do not. However, panel surveys are becoming increasingly common for epidemiological research in the social sciences [36–38]. Third, there is a possibility that people with locomotive syndrome are less likely to participate in research studies since many locomotive organ diseases, such as osteoarthritis and spinal canal stenosis, are associated with pain and limitations in movement. This could represent a potential bias. However, we used an internet survey to distribute the questionnaire in our study. Accordingly, people with locomotive syndrome could easily participate in the study, since locomotive organ diseases do not prevent their use of the internet. These days, internet users among the elderly are increasing and are becoming more common in Japan [39]. Fourth, the participants in our study were registrants with a survey company; therefore, this was not a population-based study and the participants may not be representative of the general population. Moreover, the participants received a small cash reward for participating, which may affect the randomness of the sample and induce further bias. However, the total number of registrants with the survey company used in the present study was very large, and the survey company creates the survey panels for internet questionnaires in consideration of minimizing bias. The email list used to recruit the participants for the survey was created based on random sampling from all available registers. Therefore, we consider that any bias related to randomness may be small in our study. Fifth, the cross-sectional study design is another limitation, as we could not prove causality between locomotive syndrome and the investigated lifestyle factors such as smoking and dental health. Lastly, we could not obtain quantitative data regarding the smoking status and frequency of alcohol consumption of the participants. To investigate the relationship of these factors with locomotive syndrome in greater detail, quantitative data are required.

## Conclusions

We conducted an internet panel survey to investigate the relationship between locomotive syndrome and lifestyle. Our study demonstrated an association between locomotive syndrome and lifestyle factors such as smoking and number of existing teeth, indicating that lifestyle, at least in part, might affect the development of locomotive syndrome. Accordingly, our results indicate that lifestyle modifications, such as improving oral hygiene and promoting the cessation of smoking, may represent important means to reduce the risk of locomotive syndrome. These can be achieved though addressing social, behavioral, and cultural factors in targeted communities.

## Abbreviations

CI: Confidence interval.
GLFS-25: 25-question Geriatric Locomotive Function Scale
OA: Osteoarthritis
OR: Odds ratio
